# Supplementary material for: Serum amyloid A-dependent inflammasome activation and acute injury in a mouse model of experimental stroke
Source: Res Sq. 2023 Sep 8:rs.3.rs-3258406. Preprint. [Version 1] doi: 10.21203/rs.3.rs-3258406/v1 (PMC10503850; doi:10.21203/rs.3.rs-3258406/v1)
Supplement: Supplement 1 [file NIHPPRS3258406V1-supplement-1.pdf]

Supplementary Files

This is a list of supplementary files associated with this preprint. Click to download.

- Table1.pdf
